# P2X7R Mediates the Synergistic Effect of ATP and MSU Crystals to Induce Acute Gouty Arthritis

**DOI:** 10.1155/2023/3317307

**Published:** 2023-01-12

**Authors:** Xiaoling Li, An Wan, Yiming Liu, Manyun Li, Ziwen Zhu, Chengyu Luo, Jinhui Tao

**Affiliations:** ^1^Department of Rheumatology and Immunology, The First Affiliated Hospital of USTC, Division of Life Sciences and Medicine, University of Science and Technology of China, Hefei, China; ^2^Pediatric Internal Medicine, Anhui Province Children's Hospital, Hefei, China; ^3^Department of Rheumatology and Immunology, The Affiliated Provincial Hospital of Anhui Medical University, Hefei, China

## Abstract

Activation of the nod-like receptor protein 3 (NLRP3) inflammasome by monosodium urate (MSU) crystals has been identified as the molecular basis for the acute inflammatory response in gouty arthritis. However, MSU crystals alone are not sufficient to induce acute gouty arthritis (AGA). Adenosine triphosphate (ATP) is an endogenous signaling molecule involved in the NLRP3 inflammasome activation. We aimed to explore the role of ATP in MSU crystal-induced AGA development. In peripheral blood mononuclear cell-derived macrophages obtained from gout patients, we observed a synergistic effect of ATP on MSU crystal-induced IL-1*β* release. Furthermore, in a rat model of spontaneous gout, we demonstrated that a synergistic effect of ATP and MSU crystals, but not MSU crystals alone, is essential for triggering AGA. Mechanistically, this synergistic effect is achieved through the purinergic receptor P2X7 (P2X7R). Blockade of P2X7R prevented AGA induction in rats after local injection of MSU crystals, and carrying the mutant *hP2X7R* gene contributed to the inhibition of NLRP3 inflammasome activation induced by costimulation of MSU crystals and ATP *in vitro*. Taken together, these results support the synergistic effect of ATP on MSU crystal-induced NLRP3 inflammasome activation facilitating inflammatory episodes in AGA. In this process, P2X7R plays a key regulatory role, suggesting targeting P2X7R to be an attractive therapeutic strategy for the treatment of AGA.

## 1. Introduction

Acute gouty arthritis (AGA) is classically defined as an autoinflammatory disease caused by the deposition of monosodium urate (MSU) crystals [1, 2]. Phagocytosis of MSU crystals by locally infiltrating macrophages results in the activation of the intracellular nod-like receptor protein 3 (NLRP3) inflammasome, a key process in AGA pathogenesis [3]. The activated NLRP3 inflammasome, in turn, releases mature caspase-1, which cleaves pro-IL-1*β* into biologically active IL-1*β* [4]. IL-1*β* further initiates the release of other inflammatory signals, including IL-6 and IL-8, which recruit neutrophils into the joint cavity to amplify the inflammatory response to AGA [2].

The role of MSU crystals in AGA pathogenesis is well established. However, the absence of gout in individuals with MSU crystal deposits has not been explained [5–7]. In addition to MSU crystals, soluble uric acid functions as an activator of the NLRP3 inflammasome, inducing inflammasome-dependent IL-1*β* release [8, 9]. However, the majority of patients with elevated blood uric acid are asymptomatic hyperuricemia [10]. Furthermore, hyperuricemia and MSU crystal deposition are persistent states *in vivo*, whereas AGA shows episodic manifestations. These results confirmed that neither precrystalline soluble uric acid nor postcrystalline MSU is sufficient to trigger AGA, suggesting the involvement of other predisposing factors in gout. To date, the etiology of AGA has not been well explained.

Adenosine triphosphate (ATP) is defined as a carrier of energy and a signaling molecule outside the cell [11]. Through the purinergic receptor P2X7 (P2X7R), ATP mediates the activation of the NLRP3 inflammasome playing a role in triggering the appropriate inflammatory response [12, 13]. Our previous study found that polymorphism in the human *P2X7R* (*hP2X7R*) gene was associated with gout susceptibility [14], suggesting the involvement of ATP in the pathogenesis of AGA. In addition, fluctuations in ATP levels were found to be associated with the triggers of AGA. For instance, mechanical stimulation during strenuous exercise promotes the activation of AMP-activated protein kinase, which inhibits ATP utilization and increases ATP production [15, 16]. Alcohol consumption increases the expression of mitochondrial ATP synthase in rat cardiomyocytes [17]. The adaptive thermogenic response of the body in a cold environment also increases ATP levels [18]. These findings warrant the need to study the relationship between ATP and AGA.

In the current study, we established a rat model of spontaneous gout and confirmed that the development of AGA requires the synergistic effect of ATP on MSU crystal-induced NLPR3 inflammasome activation. Furthermore, our results indicated that the synergistic effect of ATP is influenced by the function of P2X7R. Our results provided direct evidence for the involvement of ATP in AGA pathogenesis and suggested that targeting P2X7R is an attractive therapeutic strategy for AGA.

## 2. Materials and Methods

### 2.1. Animals

Male Sprague–Dawley (SD) rats weighing 200 g were purchased from the Experimental Animal Center of the First Affiliated Hospital of USTC and housed under pathogen-free conditions. Uricase knockout (*Uox-/-*) rats were constructed by Cyagen Biosciences (China) using CRISPR/Cas-mediated genome engineering techniques. The F0 generation animals were identified by PCR and sequence analysis, and germline transmission and the F1 generation were identified after mating with wild-type (WT) rats.

### 2.2. *In Vivo* Rat Model of AGA

Two rat models of AGA were used in this study, the Coderre gout model [19] and the spontaneous gout model. In the Coderre gout model, SD rats were randomly divided into three groups: (i) control group: intra-articular (IA) injection of 100 *μ*L sterile saline into the right ankle; (ii) MSU group: IA injection of 100 *μ*L MSU crystal suspension (0.1 g/mL; Sigma U2875); and (iii) MSU+Brilliant Blue G (BBG): intraperitonial (IP) administration of 500 *μ*L BBG solution (50 mg/kg; Sigma 27815) 30 minutes before IA injection of MSU crystal suspension. Ankle joint circumference and swelling index were recorded to assess the manifestation of joint inflammation in rats. The swelling index was calculated using the following formula:

(1) $$\begin{array}{c} \text{Swelling index}=\frac{\left(\text{Circumference of the treated ankle joint}-\text{Initial circumference}\right)}{\text{Initial circumference}}. \end{array}$$

The spontaneous gout model was constructed using SD and *Uox-/-* rats. In rats, uric acid is first degraded into soluble allantoin by uricase and then excreted. Hence, a hyperuricemia model was established in SD rats by IP administration of oxonic acid potassium (30 mg/100 g; Sigma 156124) for seven days. Rats with uric acid values over 180 *μ*mol/L were selected for the follow-up experiments. Next, hyperuricemic SD and *Uox-/-* rats were randomly divided into four groups: (i) control group: tail vein injection of sterile saline (100 *μ*L) for two days; (ii) MSU group: tail vein injection of MSU crystal suspension (0.1 g/mL; 100 *μ*L) for two days; (iii) ATP group: tail vein injection of ATP solution (10 mM; 200 *μ*L) for two days; and (iv) MSU+ATP group: tail vein injection of MSU crystal suspension for two days, with an injection of ATP solution on the second day after MSU crystal suspension injection (Supplementary Figure 1). Spontaneous AGA was defined as the presence of swelling and elevated skin temperature in the ankle or metatarsophalangeal joints of rats.

### 2.3. Cell Preparation and Stimulation

Human peripheral blood mononuclear cells (PBMCs) were isolated from stable gout patients using human lymphocyte isolation solution (Haoyang LTS1077) and erythrocyte lysate (Haoyang NH4CL2009). PBMCs were cultured overnight in RPMI 1640 medium (HyClone AG29775810) supplemented with 10% FBS (HyClone SV30087.03). The supernatant was discarded the following day to obtain crude macrophages. Human THP-1 cells were transfected with either empty lentivirus virus or lentivirus overexpressing WT or exon 496 mutant (E496A) *hP2X7R* gene (Hanvon Biotech Co., Ltd., China) in the presence of polybrene (Hanking Biotech). The cells were then cultured in RPMI 1640 medium supplemented with 10% FBS for 72 h. Subsequently, the transfected THP-1 cells (5 × 10^4^ cells/well) were induced to differentiate overnight with phorbol myristate acetate (PMA, 100 ng/mL; Sigma P1585).

Cells were stimulated with LPS (50 ng/mL; for priming NLRP3 inflammasome activation; Invitrogen LPS-EK) for 3 h. Then, either MSU (100 *μ*g/mL, 4 h), ATP (1 mM, 0.5 h; Sigma A6559), or nigericin (10 *μ*M; 0.5 h; MCE HY-100381) was added to the culture to activate the NLRP3 inflammasome, according to the arrangement of the experimental groups.

### 2.4. Enzyme-Linked Immunosorbent Assay (ELISA)

ELISA was performed to measure IL-1*β* levels in culture supernatants (NOVUS Biological VAL101) and rat serum (Multi Sciences A301BH10152), as per the manufacturer's instructions.

### 2.5. Quantitative PCR (qPCR)

RNA and cDNA were extracted from stimulated THP-1 cells and peripheral whole blood cells of the rat models using a HiPure Blood RNA Kit (Solarbio R4161-02) and cDNA Synthesis Kit (BIOER BSB40M1). qPCR was performed to quantify the expressions of *NLRP3*, *caspase-1*, and *IL-1β* mRNA with SYBR Green PCR Master Mix (Muma A4004M). The primer sequences are listed in Supplementary Table 1.

### 2.6. Western Blot

Western blotting (WB) was performed to detect the expressions of NLRP3, caspase-1, and IL-1*β* proteins in joint grinds from the rat model. Protein lysates were extracted using RIPA buffer (Epizyme Biomedical PC101) and protease inhibitor (Epizyme Biomedical GRF101). Electrophoresis was performed under a segmented constant voltage (stacking gel, 80 V; voltage gel, 120 V). The bands were transferred to a membrane in an ice bath with a constant current (200 mA). The membrane was then blocked with 5% skim milk powder for 1 h. The following antibodies were used for probing overnight at 4°C: IL-1*β* (1 : 1000, CST D3A3Z), caspase-1 (1 : 1000, CST D7F10), NLRP3 (1 : 1000, CST D4D8T), and GAPDH (1 : 1000, CST D16H11). The membrane was then incubated with goat anti-rabbit IgG-HRP (1 : 5000, Abbkine A21010) for 1 h at room temperature. The results were scanned using chemiluminescence detection with TanonFine-DoX6 (Tianneng).

### 2.7. Confocal Imaging

The joints of the spontaneous gout rat model were placed in a cryo-embedding medium and quickly extracted with liquid nitrogen to make frozen sections. They were then incubated with tissue-covered primary antibodies against CD3 (Abcam Ab5690) and HIS48 (Santa Cruz Sc19613) overnight at 4°C. Then, they were incubated with the secondary antibody for 1 hour at room temperature. The nuclei were stained with DAPI working solution and incubated for 15 min at room temperature. The neutral gel was added dropwise to seal the sections, and the sections were photographed and recorded under a confocal microscope (ZEISS LSM980).

### 2.8. Dye Uptake Assay

HEK-293T cells were cultured for 24 hours (1 × 10^5^ cells/mL) in DMEM (Thermo 11965118) supplemented with 10% FBS, followed by transfection with lentivirus for 24 h in the presence of polybrene. Cells were suspended in HEPES buffered medium (10 mM HEPES, 150 mM KCl, 5 mM D-glucose, and 0.1% bovine serum albumin). Then, 25 *μ*M ethidium bromide (EB; Sigma 1239-45-8) and ATP (1 mM; Sigma A6559) were added to the flow tube, and the cells were collected every 5 seconds using a CytoFLEX flow cytometer (Beckman). The average fluorescence intensity of each sample was read using CytExpert.

### 2.9. Sequencing of the Rat *P2X7R* Gene

Rat peripheral blood DNA was extracted for full coding exon sequencing of the *P2X7R* gene. Primers were designed using the Primer3 software. PCR products were purified by shrimp alkalase (Promega MT0093) and exonuclease I (Epicenter 60684050) and sequenced with a BigDye3.1 kit (ABI 4337455). Sequencing reactions were purified by alcohol and then sampled on a sequencer (ABI 3730XL). After sequencing, a comparison with the rat *P2X7R* (*rP2X7R*) genome in the UCSC database revealed a total of 27 loci with base mutations. The loci with large differences in allele frequencies between the AGA and non-AGA groups were screened for linkage disequilibrium locus testing (*rP2X7R* gene loci 33514, 25624, 1333, 24771, 17845, 16703, and 1016) (Supplementary Figure 2). Finally, *rP2X7R* loci 954, 1016, and 33514 were identified for analysis.

### 2.10. Ethics Statement

The study protocols for the collection and processing of animal and human samples were approved by the Animal Ethics Committee (2021-N(A)-041) and Research Ethics Committee (2021KY Lun Review No. 162) of the First Affiliated Hospital of USTC (Anhui Provincial Hospital), respectively. All patients gave their informed consent before their inclusion in the study.

### 2.11. Statistical Analyses

Data were analyzed using IBM SPSS Statistics for Windows, version 17.0 (IBM Corp, Armonk). Quantitative data were tested for normality. Independent sample *t*-test and one-way analysis of variance were used to compare differences between two groups and multiple groups, respectively. Dunnett's *t*-test was used to validate the significant differences revealed by one-way analysis of variance. Statistical significance was set at *P* < 0.05. The Hardy-Weinberg equilibrium (HWE) of the genotype distribution was analyzed using the *χ*^2^ test. Group comparisons of constituent ratios were performed using the *χ*^2^ test or the adjusted *χ*^2^ test and expressed as odds ratios (ORs) and 95% confidence intervals (95% CIs). The inspection level is *χ*^2^ = 0.05. All bar graphs were plotted using the GraphPad Prism 8.0 software (GraphPad Software).

## 3. Results

### 3.1. ATP and MSU Crystals Act Synergistically on IL-1*β* Release *In Vitro*

**Figure 1:**
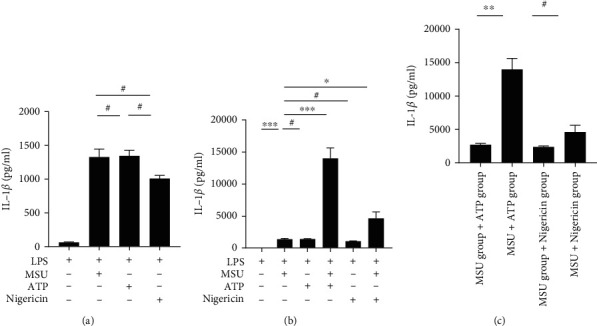
Synergistic effect of ATP and MSU crystals on the release of IL-1*β*. ELISA of IL-1*β* in the supernatants of the culture of PBMC-derived macrophages obtained from gout patients. (a) LPS (50 ng/mL)-primed cells stimulated with MSU crystal suspension (100 *μ*g/mL), ATP (1 mM), and nigericin (10 *μ*M). (b) LPS-primed cells were stimulated with MSU, ATP, MSU+ATP, nigericin, or MSU+nigericin. (c) Comparison of IL-1*β* levels in different groups in (b). Statistics were analyzed using the SNK-*q* test (a), Dunnett's *t*-test (b), and independent sample *t*-test (c). Data are presented as mean ± SEM. ^∗^*P* < 0.05; ^∗∗^*P* < 0.01; ^∗∗∗^*P* < 0.001.

A preponderance of evidence supports the notion that AGA is a multifactorial disorder [20]. MSU crystal deposition is a necessary but not sufficient trigger for the disease. To determine whether ATP is involved in AGA pathogenesis, the effect on IL-1*β* secretion was compared in PBMC-derived macrophages obtained from gout patients stimulated with MSU crystals, ATP, and nigericin. ELISA results showed no significant differences in IL-1*β* release under stimulation by different NLRP3 activators alone (Figure 1(a)). Given that MSU crystals underlie the pathogenesis of AGA, the effect of ATP and nigericin on IL-1*β* secretion when coacting with MSU crystals was further evaluated. The results showed that ATP significantly enhanced MSU crystal-induced IL-1*β* secretion (Figure 1(b)). More importantly, the levels of IL-1*β* after costimulation with ATP and MSU crystals were significantly higher than the cumulative levels of IL-1*β* obtained after their solo stimulations (Figure 1(c)). These findings imply that ATP and MSU crystals exert a synergistic effect on the release of IL-1*β*, while nigericin and MSU crystals exert an additive effect. Furthermore, even with increasing stimulatory concentrations of nigericin, its combined effect with MSU crystals was still lower than that of ATP (Figure S3). These results indicated that ATP synergizes with MSU crystals to stimulate the activation of the NLRP3 inflammasome and the subsequent release of IL-1*β* in gout.

### 3.2. ATP and MSU Crystals Synergistically Induce AGA in Rats

**Figure 2:**
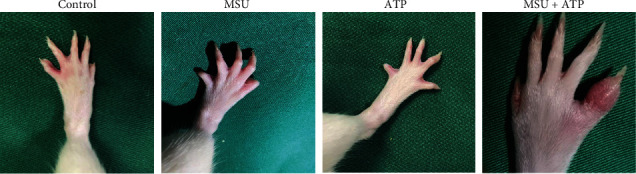
Manifestation of spontaneous arthritis in rats. Representative images of rat joints after the injection of sterile saline, MSU, ATP, and MSU+ATP in the tail vein of hyperuricemic SD rats (12 h). Spontaneous arthritis manifested as redness and swelling in the toe joints of the rats.

Since ATP and MSU crystals exert synergistic effects on IL-1*β* secretion *in vitro*, we hypothesized that the combined action of ATP and MSU crystals might contribute to the induction of AGA *in vivo*. To test this hypothesis, a rat model of spontaneous arthritis was constructed by tail vein injection of the MSU crystals with or without ATP. The results showed that some hyperuricemic SD rats developed spontaneous arthritis, characterized by redness and swelling of the toe joints (Figure 2). Among them, the incidence of arthritis was 5% (2/40) in the MSU group and 30% (12/40) in the MSU+ATP group, while no rats in either the control or the ATP group developed arthritis (both 0/40). There was a significant difference in AGA incidence among the four groups (*χ*^2^ = 30.199, *P* < 0.001). Subsequently, the same analysis was performed with *UOX*^−/−^ rats. Consistent with the results observed in SD rats, the prevalence of arthritis in the MSU+ATP group was 38.46% (5/13). However, none of the rats in the control group (0/5), the MSU group (0/5), or the ATP group (0/5) developed arthritis. AGA incidence was significant differences among the four groups (*χ*^2^ = 8.953, *P* = 0.03). The above results suggested that MSU stimulation alone is insufficient to induce a significant level of arthritis and that the development of spontaneous arthritis in rats requires combined stimulation by ATP and MSU crystals.

**Figure 3:**
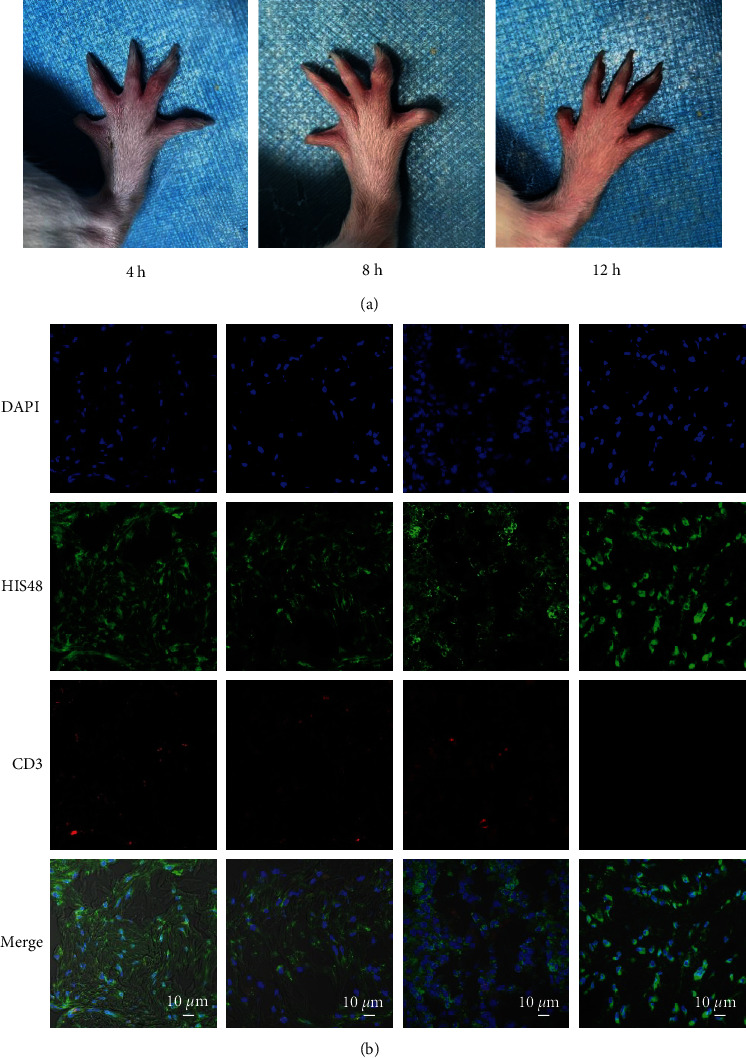
Characteristics of spontaneous arthritis in rats. (a) Manifestation of spontaneous arthritis in rats over time. Rats showed redness and swelling of the fourth metatarsophalangeal joint 4 h after tail vein injection of MSU+ATP; joint swelling increased 8 h after the injection; joint symptoms further increased 12 h after the injection, along with an increase in the skin temperature. (b) Representative confocal microscopy images of joint sections of rats with spontaneous arthritis, showing infiltration of neutrophils (HIS48, green) and T cells (CD3, red).

It was observed that the symptoms of spontaneous arthritis induced by the combined action of MSU crystals and ATP peaked in arthritic symptoms 12 h after injection (Figure 3(a)). In addition, confocal microscope images showed that the local inflammatory cell infiltration in the affected joint was dominated by neutrophils (HIS48), accounting for more than 90% of the infiltrated cells, while a limited quantity of lymphocytes (CD3) was present (Figure 3(b)). These manifestations are similar to the features of human gout pathogenesis, suggesting that the synergistic action of ATP and MSU crystals induces spontaneous arthritis in rats as a gout flare.

### 3.3. ATP and MSU Crystals Synergistically Induce AGA in Rats by Fully Activating the NLRP3 Inflammasome

**Figure 4:**
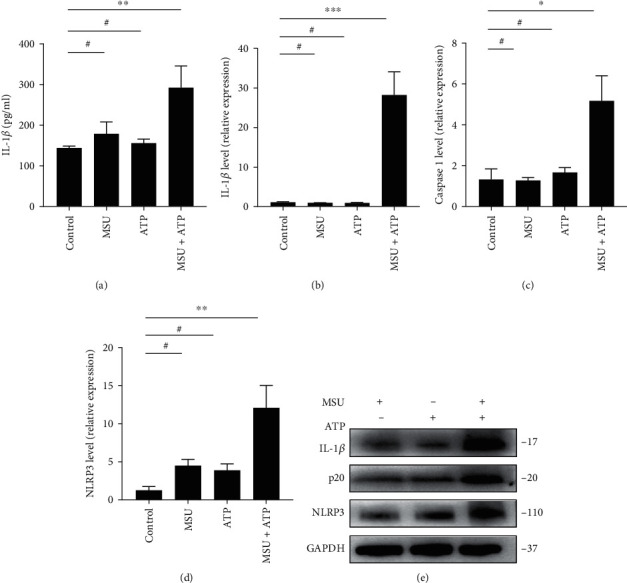
ATP and MSU crystals induced NLRP3 inflammasome activation in rats. (a) ELISA of serum IL-1*β* levels in rats. (b–d) qPCR of *IL-1β*, *caspase-1*, and *NLRP3* mRNA levels in peripheral whole blood cells of rats. (e) WB of IL-1*β*, caspase-1, and NLRP3 protein levels in the joints and surrounding tissues of rats. Dunnett's *t*-test was used for statistical analysis. Data are expressed as mean ± SEM. *n* = 3-8. ^∗^*P* < 0.05; ^∗∗^*P* < 0.01; ^∗∗∗^*P* < 0.001.

Given that NLRP3 inflammasome activation is a hallmark of AGA, we examined the levels of IL-1*β*, caspase-1, and NLRP3 in each group of the spontaneous gout rat model. As detected by ELISA, serum IL-1*β* levels were significantly higher in the MSU+ATP group of AGA rats (Figure 4(a)). Similarly, the qPCR and WB results showed that the expressions of IL-1*β*, caspase-1, and NLRP3 were higher in the MSU+ATP group rats than in the remaining groups (Figures 4(b)–4(e)). The above results suggested that ATP and MSU crystals act synergistically to initiate episodes of inflammation in AGA models by fully activating the NLRP3-caspase-1 signaling pathway to release adequate levels of IL-1*β*.

### 3.4. The Synergistic Effect of ATP and MSU Crystals Is Affected by the Function of P2X7R

#### 3.4.1. Inhibition of P2X7R Alleviates MSU Crystal-Induced AGA in Rats

**Figure 5:**
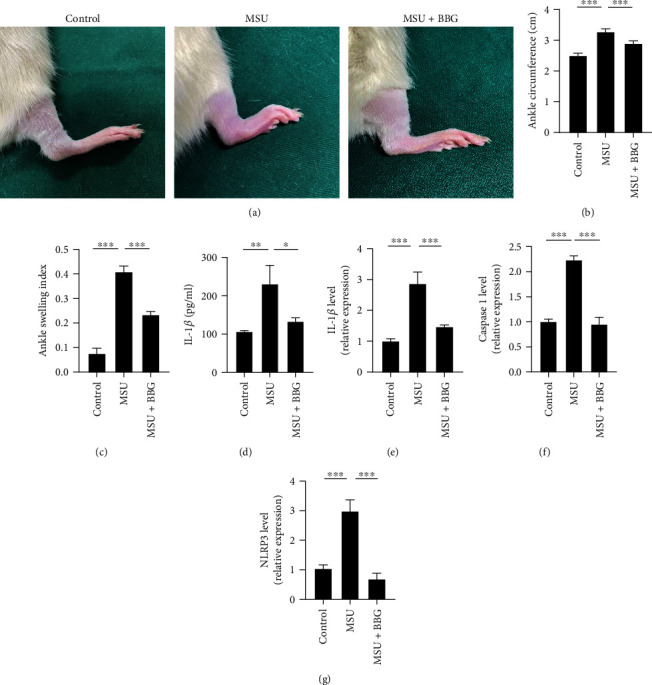
Inhibition of P2X7R alleviates MSU crystal-induced AGA in rats. Coderre gout models were divided into the control group, MSU group, and MSU+BBG group. (a) Representative images of the right ankle joint of each group of rats after treatment (12 h). (b, c) Right ankle joint circumference and joint swelling index of rats (12 h). (d) ELISA of serum IL-1*β* levels in rats. (e–g) qPCR of *IL-1β*, *caspase-1*, and *NLRP3* mRNA levels in the peripheral whole blood cells of rats. Dunnett's *t*-test was used for statistical analysis. Data are expressed as mean ± SEM. *n* = 4. ^∗^*P* < 0.05; ^∗∗^*P* < 0.01; ^∗∗∗^*P* < 0.001.

We already demonstrate the contributory role of ATP in a rat model of spontaneous gout; however, AGA induced by the local injection of MSU crystals does not appear to require ATP signaling in the traditional Coderre model of gout. To determine the involvement of ATP in AGA, BBG treatment was employed to inhibit the function of P2X7R to block ATP signaling in the Coderre model. As expected, MSU crystal-induced local gout symptoms in the ankle joint of rats were effectively relieved by BBG treatment, as indicated by a reduction in the degree of joint swelling (Figures 5(a)–5(c)). We further examined the effect of BBG treatment on IL-1*β* levels and NLRP3 inflammasome activation in rats with MSU crystal-induced AGA. Our results showed that BBG treatment significantly inhibited the overexpression of IL-1*β*, caspase-1, and NLRP3 (Figures 5(d)–5(g)), indicating that targeting P2X7R to block the ATP-induced NLRP3-caspase-1-IL-1*β* signaling pathway alleviates MSU crystal-induced gout inflammation in rats. This finding suggested that ATP signaling exerted a synergistic effect in AGA induced by the local injection of MSU crystals.

#### 3.4.2. Polymorphism of the rP2X7R Gene Locus Affects the Synergistic Effect of ATP in Rat Gout Models

As observed, the synergistic effect of ATP and MSU crystals was responsible for a spontaneous gout incidence of ~30% in rats. It is suggested that the involvement of ATP in gout might be influenced by the function of P2X7R. However, the *P2X7R* gene has multiple polymorphisms, which significantly alter the function of the protein. To determine the relationship between *rP2X7R* locus polymorphisms and AGA, the full coding exons of the *rP2X7R* gene were sequenced in a rat model of spontaneous gout. Compared with the *rP2X7R* genome in the UCSC database, a total of 27 gene loci were found to carry base mutations. Loci with large differences in allele frequencies between the AGA and AGA-free groups (20 vs. 25) were screened and subjected to linkage disequilibrium analysis. Ultimately, *rP2RX7* gene loci 954, 1016, and 33514 were selected for subsequent analysis. First, the HWE balance test was performed to assess the genotype distribution of these three loci of the *rP2X7R* gene. The genotypes at locus 954 were AA, AC, and CC; those at locus 1016 were AA, AG, and GG; and those at locus 33514 were CC, CT, and TT. The genotypic distribution of the loci was in accordance with the HWE balance (Table 1).

Analysis of allele frequencies and genotype distributions revealed that the frequencies of alleles A and G at locus 1016 of the *rP2X7R* gene were 60% (30/50) and 40% (20/50) in the AGA-free group and 82.5% (33/40) and 17.5% (7/40) in the AGA group, respectively. For this locus, the frequency of allele A in the AGA group was significantly higher than that in the AGA-free group (*χ*^2^ = 5.357, *P* = 0.021). The distribution of genotypes AA, AG, and GG at locus 1016 was 32% (8/25), 56% (14/25), and 12% (3/25) in the AGA-free group and 70% (14/20), 25% (5/20), and 5% (1/20) in the AGA group, respectively. The genotype distribution was significantly different between the two groups (*χ*^2^ = 6.586, *P* = 0.037). However, the allele frequencies and genotype distribution at the *rP2X7R* loci 954 and 33514 were not found to be significantly different between these two groups of rats (Table 2).

The above results showed that allele A and genotype AA at the 1016 loci of the *rP2X7R* gene were associated with the onset of gout in rats, implying that the synergistic effect of ATP and MSU crystals on AGA induction in rats is significantly affected by the *rP2X7R* gene polymorphism.

#### 3.4.3. Loss of hP2X7R Function Impairs the Synergistic Effect of ATP *In Vitro*

**Figure 6:**
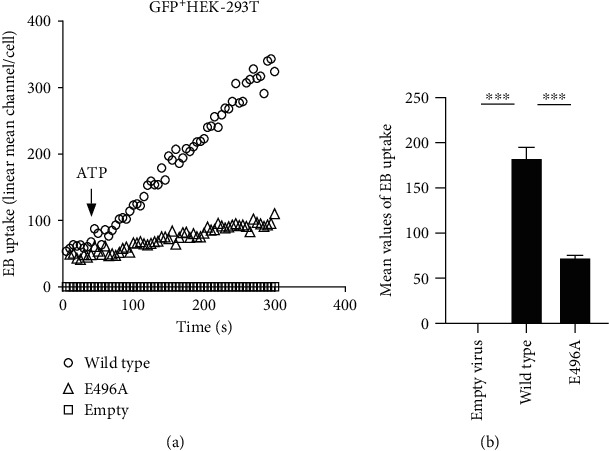
The E496A mutant inhibits ATP-induced P2X7R pore formation. HEK-293T cells were transfected with lentiviruses overexpressing WT and E496A mutant *hP2X7R* genes as well as empty lentiviruses. (a) Flow cytometry results of P2X7R-dependent EB uptake induced by 1 mM ATP (indicated by arrow) in HEK-293T cells overexpressing different lentiviruses. (b) Mean values of EB uptake in the three groups. Dunnett's *t*-test was used for statistical analyses. Data are expressed as mean ± SEM. ^∗∗∗^*P* < 0.001.

We found that the *rP2X7R* gene locus polymorphism is associated with the induction of AGA by the synergistic action of ATP and MSU crystals. To verify whether the *hP2X7R* gene polymorphism also affected this synergistic effect, first, the relationship between the *hP2X7R* gene polymorphisms and corresponding protein function was assessed. P2X7R is an ATP-gated channel receptor that, upon sustained stimulation, forms pores that allow macromolecules to enter cells. WT and E496A mutant of the *hP2X7R* genes were transfected into HEK-293T cells to assess the effect on ATP-induced P2X7R pore formation. Flow cytometry showed that cells overexpressing the WT *hP2X7R* gene showed a significant increase in EB uptake after ATP stimulation, while cells overexpressing the E496A mutant effectively inhibited ATP-induced EB uptake (Figure 6). This result suggested that E496A *hP2X7R* gene inhibits ATP-induced P2X7R pore formation and attenuates P2X7R's function.

**Figure 7:**
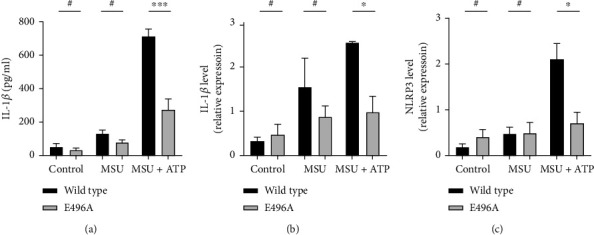
The effect of NLRP3 inflammasome activation in THP-1 cells overexpressing the *hP2X7R* gene of the E496A mutant. Lentiviruses overexpressing the WT and E496A *hP2X7R* genes were transfected into THP-1 cells receiving stimuli of LPS (control group), MSU (MSU group), or MSU+ATP (MSU+ATP group). (a) ELISA of IL-1*β* levels in THP-1 cells after different stimulations. (b, c) qPCR of *IL-1β* and *NLRP3* mRNA levels in THP-1 cells after different stimulations. Independent sample *t*-test was used for statistical analysis. Data are expressed as mean ± SEM. ^∗^*P* < 0.05; ^∗∗∗^*P* < 0.001.

To assess whether suppressed P2X7R protein function impaired the synergistic effects of ATP and MSU crystals *in vitro*, THP-1 cells overexpressing WT and E496A mutant *hP2X7R* genes were stimulated with MSU+ATP. As expected, the supernatant IL-1*β* levels of THP-1 cells expressing the *hP2X7R* gene E496A mutant were significantly lower than those of the WT *hP2X7R* gene-expressing cells after stimulation with MSU+ATP (Figure 7(a)). Importantly, the mRNA expression levels of the *IL-1β* and *NLRP3* genes also showed similar trends (Figures 7(b) and 7(c)). When stimulated with MSU crystals alone, there was no significant difference in IL-1*β* levels of THP-1 cells transfected with the WT virus and mutant virus. These results indicated that the synergistic effect of ATP and MSU crystals is influenced by hP2X7R's function *in vitro*.

## 4. Discussion

Although ATP and MSU crystals can act together to activate the NLRP3 inflammasome to induce AGA, several other stimulatory signals also activate the NLRP3 inflammasome, including nigericin, glucose, and lipids. It is unclear why only ATP plays an important regulatory role in the pathogenesis of gout. In the present study, the effects of different NLRP3 inflammasome activators on IL-1*β* secretion were evaluated *in vitro* using the PBMC-derived macrophages obtained from gout patients. Among the conventional NLRP3 activators, ATP exerted a synergistic effect on MSU crystal-induced IL-1*β* secretion, whereas nigericin exerted an additive effect. On the basis of these results, a spontaneous gout rat model mimicking human gout onset was constructed for *in vivo* analysis. We demonstrated that MSU crystal stimulation alone is not sufficient to induce a significant degree of AGA. In contrast, costimulation with ATP sufficiently activated the NLRP3 inflammasome, leading to spontaneous gout onset in rats. Our data suggested that the episodes of inflammation of AGA require the synergistic effect of ATP on MSU crystal-induced NLRP3 inflammasome activation.

The specific mechanisms underlying NLRP3 inflammasome activation are not well understood. The three main hypotheses currently proposed are potassium efflux, mitochondrial reactive oxygen species, and lysosomal rupture [21]. Among them, alteration in intracellular potassium ion concentration is the earliest discovered and most necessary signal for NLRP3 inflammasome activation [22, 23]. MSU crystals, ATP, and nigericin induce potassium ion efflux via different mechanistic pathways. Phagocytosed MSU crystals function by mediating the rupture of phagocytic lysosomes in cells to activate water channel proteins, leading to cell swelling and a decrease in intracellular potassium ion levels [24]. Nigericin promotes potassium ion/hydrogen ion exchange across the mitochondrial membrane to activate NLRP3 inflammasomes [22]. ATP acts on the ion channel receptor P2X7R. In addition to causing potassium ion efflux, it also promotes intracellular and extracellular calcium ion and sodium ion influx [25]. Calcium ions, on the one hand, activate the NLRP3 inflammasome by promoting the spontaneous binding of NLRP3 and ASC. On the other hand, large amounts of calcium ions lead to mitochondrial damage manifested by the generation of mitochondrial reactive oxygen species, which is another important factor in the activation of the NLRP3 inflammasome [26, 27]. Thus, the regulation of multiple ion fluxes by ATP might be responsible for its synergistic role with MSU crystals in the pathogenesis of gout.

The role of ATP was confirmed in a rat model of spontaneous gout. However, in the conventional Coderre gout model, AGA induction by injection of only MSU crystals into the ankle joint indicates that MSU crystals are sufficient for AGA development, lacking any evidence of ATP's involvement. However, pyroptosis was observed when MSU crystals induced IL-1*β* secretion [28, 29]. The release of ATP from pyroptosis into the extracellular is then involved in the pathogenesis of gout [30]. Our study showed that inhibition of P2X7R's function to block ATP-mediated activation of the NLRP3 inflammasome attenuated MSU crystal-induced AGA in rats, confirming the significance of ATP-P2X7R signaling in MSU crystal-induced AGA. This result was consistent with an *in vitro* study by Marinho et al., which reported that MSU crystal stimulation-induced IL-1*β* secretion was dependent on P2X7R's activation, an effect that corresponds to the extracellular concentration of ATP [31].

P2X7R is an ATP-gated channel receptor. Several recent studies have revealed that *rP2X7R* gene polymorphisms affect protein function [32, 33]; however, its relationship with gout is limited. In the current study, exon sequencing of the *rP2X7R* gene in AGA and AGA-free rats in a spontaneous gout model revealed that an increased frequency of allele A and genotype AA at locus 1016 was associated with the development of gout. Locus 1016 of the *rP2X7R* gene is located in the 5′UTR rather than the gene coding region. We speculate that base changes might modulate the function of rP2X7R by affecting the stability or translation of the corresponding mRNA. Knowledge about locus 1016 of the *rP2X7R* gene is still limited and warrants further exploration. The sequence homology between rat and human *P2X7R* genes is 80% [25]. Our previous population studies have already demonstrated that *hP2X7R* gene polymorphism is associated with gout susceptibility [14]. By constructing lentiviruses overexpressing the *hP2X7R* gene and transfecting them in 293T cells, we confirmed the downregulation of P2X7R function in the E496A mutant, which was consistent with the previous studies [34]. However, some studies have demonstrated an elevated IL-1*β* release after ATP stimulation of whole blood cells in gout patients carrying the E496A mutant compared to the normal population [35]. Furthermore, in the current study, the THP-1 cells overexpressing the E496A mutant *hP2X7R* gene, after being subjected to costimulation with ATP and MSU crystals, showed diminished levels of NLRP3 inflammasome activation and IL-1*β* release. This finding suggested that the loss of function of P2X7R is detrimental to the synergistic effects of ATP and MSU crystals, which might help explain the lack of gout development in some hyperuricemic patients.

We found evidence for the involvement of ATP in the induction of AGA. The remission of AGA might also be associated with the breakdown of ATP during the inflammatory response. After a gout flare, the expressions of CD39 and CD73 increase in the inflammatory environment, and ATP signaling is terminated by progressive conversion to AMP and adenosine [36, 37]. In contrast to ATP and ADP, the binding of adenosine to P1 receptors has been found to exert an inhibitory effect on inflammation *in vivo* [38]. Thus, the interplay between different intracellular purinergic signaling pathways, which together regulate gout flare and remission, contributes to gout being a self-limiting inflammatory disease.

## 5. Conclusion

In conclusion, we provided evidence that the synergistic effect of ATP on MSU crystal-induced NLRP3 inflammasome activation is required for AGA development in rats in the context of hyperuricemia. The present study further demonstrates that *P2X7R* gene polymorphism and function influence the synergistic effect of ATP in this context. Thus, targeting P2X7R might be a promising therapeutic approach for the treatment of AGA.

## Figures and Tables

**Table 1:** HWE balance test of the *rP2X7R* gene loci in AGA and AGA-free rats.

Locus/group	Genotype			HWE	
954	AA	AC	CC	*χ* ^2^	*P*
AGA-free	6	16	3	2.230	0.135
AGA	10	6	4	2.321	0.128

1016	AA	AG	GG	*χ* ^2^	*P*
AGA-free	8	14	3	0.694	0.404
AGA	14	5	1	0.36	0.548

33514	CC	CT	TT	*χ* ^2^	*P*
AGA-free	24	1	0	0.010	0.919
AGA	16	4	0	0.247	0.619

**Table 2:** Allele and genotype frequency distribution of the *rP2X7R* gene in AGA and AGA-free rats.

		AGA-free (*n* (%))	AGA (*n* (%))	*χ* ^2^	*P*	OR (95% CI)
*954*						
Allele	A	28 (51.9)	26 (48.1)	0.75	0.386	0.685 (0.291-1614)
	C	22 (61.1)	14 (38.9)			
Genotype	AA	6 (24)	10 (50)	5.314	0.07	—
	AC	16 (64)	6 (30)			
	CC	3 (12)	4 (20)			

*1016*						
Allele	A	30 (60)	33 (82.5)	5.357	0.021	0.426 (0.16-1.139)
	G	20 (40)	7 (17.5)			
Genotype	AA	8 (32)	14 (70)	6.586	0.037	—
	AG	14 (56)	5 (25)			
	GG	3 (12)	1 (5)			

*33514*						
Allele	C	49 (98)	36 (90)	2.81	0.094	5.444 (0.584-50.792)
	T	1 (2)	4 (10)			
Genotype	CC	24 (96)	16 (80)	2.982	0.084	6 (0.613-58.706)
	TT	1 (4)	4 (20)			

## Data Availability

The data presented in this study are available on request from the corresponding author.

## Supplementary Materials

Supplementary Materials Figure S1: process of spontaneous gout in the SD rat model. Figure S2: linkage disequilibrium analysis of the *rP2X7R* gene loci. Figure S3: synergistic effect of ATP and MSU crystals on the release of IL-1*β*. Table S1: the sequences of the primers.
