# Supplementary material for: P2X7R Mediates the Synergistic Effect of ATP and MSU Crystals to Induce Acute Gouty Arthritis
Source: Oxid Med Cell Longev. 2023 Jan 12;2023:3317307. doi: 10.1155/2023/3317307 (PMC9851801; doi:10.1155/2023/3317307)
Supplement: Supplementary Materials — Figure S1: process of spontaneous gout in the SD rat model. Figure S2: linkage disequilibrium analysis of the rP2X7R gene loci. Figure S3: synergistic effect of ATP and MSU crystals on the release of IL-1β. Table S1: the sequences of the primers. [file 3317307.f1.docx]

**Supplementary Data**


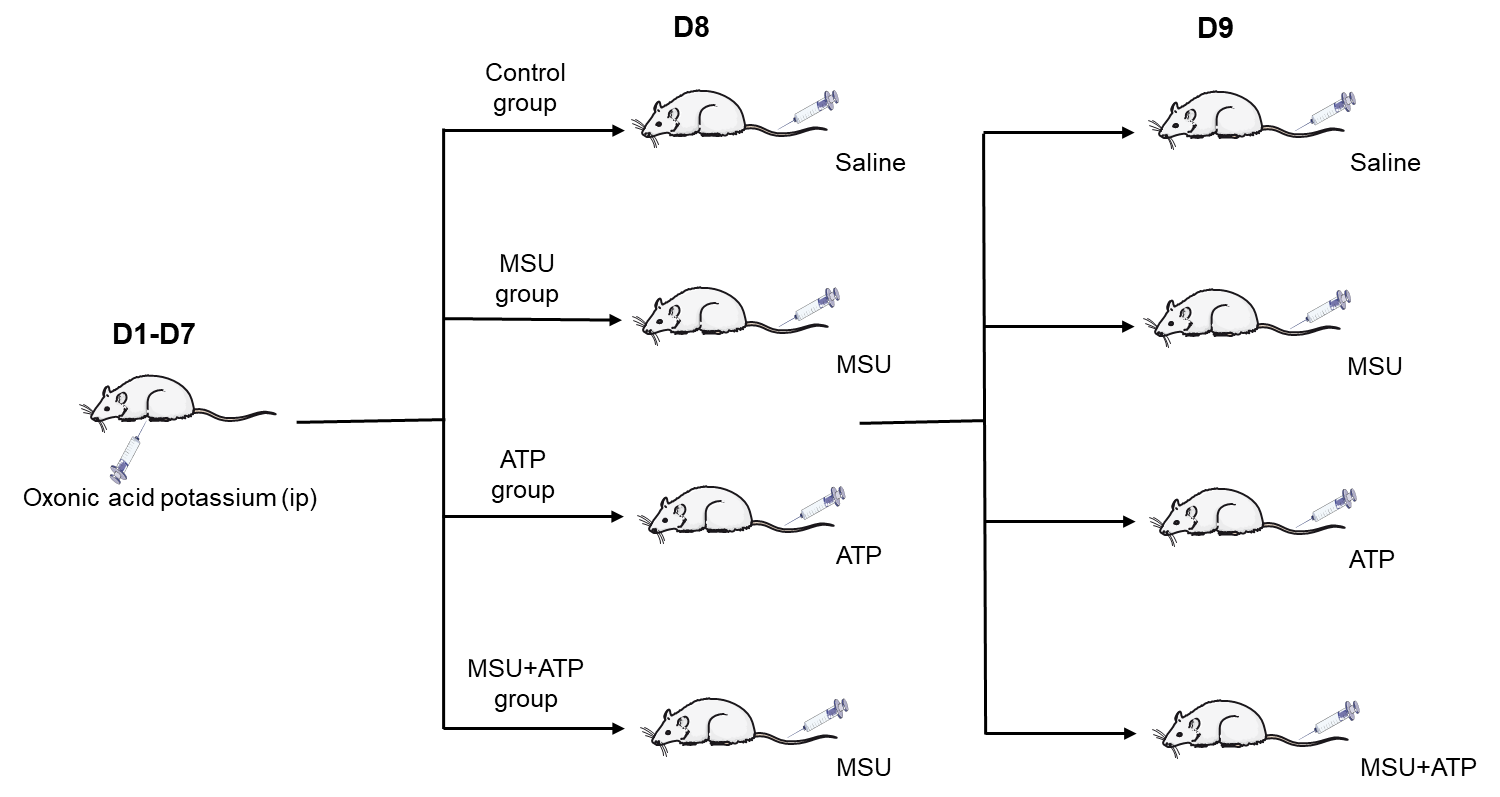


**Figure S1 Process of spontaneous gout in the SD rat model**

On days 1-7, SD rats were injected intraperitoneally with oxonic acid potassium to construct a rat model of hyperuricemia. The hyperuricemic rat model was divided into four groups to receive different treatments. Control group: Day 8-9, tail vein injection of saline; MSU group: Day 8-9, tail vein injection of MSU crystals suspension; ATP group: Day 8-9, tail vein injection of ATP solution; MSU+ATP group: Day 8-9, tail vein injection of MSU crystals suspension, followed by ATP solution on the second day after MSU crystals suspension injection.


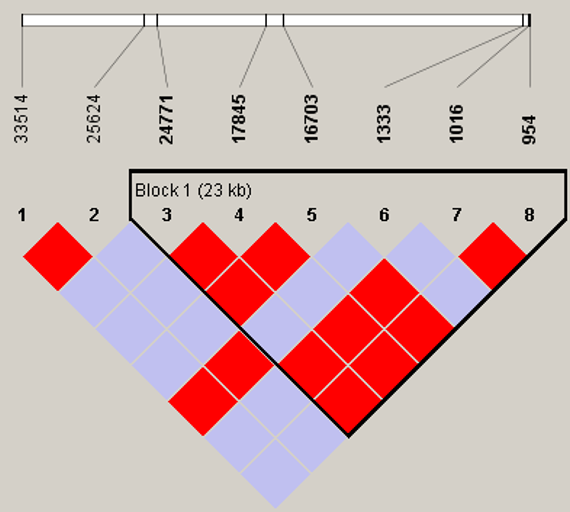


**Figure S2** **Linkage disequilibrium analysis of the *rP2X7R* gene loci**

The linkage disequilibrium test showed that *rP2X7R* gene loci 33514, 25624, 1333, r^2^>0.8; loci 24771, 17845, 16703, 1016, r^2^>0.8.

## Table S1 The sequences of the primers

| Gene | F-seq（5’-3’） | R-seq（5’-3’） |
| --- | --- | --- |
| rat-*Actβ* | ACCCGCCACCAGTTCGC | CACGATGGAGGGGAAGACG |
| rat-*nlrp3* | GTGGAGATCCTAGGTTTCTCTG | CAGGATCTCATTCTCTTGGATC |
| rat-*casp1* | GAAACGCCATGGCTGACAAG | CATGATCGCACAGGTCTCGT |
| rat-*IL-1β* | TGGCAACTGTCCCTGAACTC | AAGGGCTTGGAAGCAATCCTTA |
| h-*Actβ* | CCTTCCTGGGCATGGAGTCCTG | GGAGCAATGATCTTGATCTTC |
| h-*nlrp3* | GATCTTCGCTGCGATCAACAG | CGTGCATTATCTGAACCCCAC |
| h-*casp1* | AATAAATGGCTTGCTGGATGAG | CCTCCTGGTCCTGAAGATGC |
| h-*IL-1β* | TCATTGTGGCTGTGGAGAAG | AGGCCACAGGTATTTTGTCG |

**Figure S3 Synergistic effect of ATP and MSU crystals on the release of IL-1β**

LPS-primed cells stimulated with MSU, MSU+ATP, MSU+nigericin (10 μM), or MSU+nigericin (100 μM). Statistics were analyzed using the Dunnett’s t-test. Data are presented as the mean ± SEM. ****P*<0.001.
